# *GRIN2B* Mutations in West Syndrome and Intellectual Disability with Focal Epilepsy

**DOI:** 10.1002/ana.24073

**Published:** 2014-01-02

**Authors:** Johannes R Lemke, Rik Hendrickx, Kirsten Geider, Bodo Laube, Michael Schwake, Robert J Harvey, Victoria M James, Alex Pepler, Isabelle Steiner, Konstanze Hörtnagel, John Neidhardt, Susanne Ruf, Markus Wolff, Deborah Bartholdi, Roberto Caraballo, Konrad Platzer, Arvid Suls, Peter De Jonghe, Saskia Biskup, Sarah Weckhuysen

**Affiliations:** 1 Division of Human Genetics, University Children’s Hospital InselspitalBern, Switzerland; 2 Partners of EuroEPINOMICS, RES consortium; 3 Neurogenetics Group, Department of Molecular Genetics, Vlaams Institute of BiotechnologyAntwerp, Belgium; 4 Laboratory of Neurogenetics, Institute Born-Bunge, University of AntwerpAntwerp, Belgium; 5 Department of Neurophysiology and Neurosensory Systems, Technical University DarmstadtDarmstadt, Germany; 6 Biochemistry III, Faculty of Chemistry, University of BielefeldBielefeld, Germany; 7 Department of Pharmacology, University College London School of PharmacyLondon, United Kingdom; 8 CeGaT GmbHTübingen, Germany; 9 Institute of Medical Molecular Genetics, University of ZurichSwitzerland; 10 Department of Neuropediatrics, University of TübingenTübingen, Germany; 11 Institute of Clinical GeneticsKlinikum Stuttgart, Stuttgart, Germany; 12 Department of Neurology, Juan P. Garrahan Pediatric HospitalBuenos Aires, Argentina; 13 Department of Human Genetics, University of LübeckLübeck, Germany; 14 Department of Neurology, Antwerp University HospitalAntwerp, Belgium; 15 Hertie Institute of Clinical Brain Research and German Center for Neurodegenerative Diseases, University of TübingenTübingen, Germany

## Abstract

**Objective:**

To identify novel epilepsy genes using a panel approach and describe the functional consequences of mutations.

**Methods:**

Using a panel approach, we screened 357 patients comprising a vast spectrum of epileptic disorders for defects in genes known to contribute to epilepsy and/or intellectual disability (ID). After detection of mutations in a novel epilepsy gene, we investigated functional effects in *Xenopus laevis* oocytes and screened a follow-up cohort.

**Results:**

We revealed de novo mutations in *GRIN2B* encoding the NR2B subunit of the N-methyl-D-aspartate (NMDA) receptor in 2 individuals with West syndrome and severe developmental delay as well as 1 individual with ID and focal epilepsy. The patient with ID and focal epilepsy had a missense mutation in the extracellular glutamate-binding domain (p.Arg540His), whereas both West syndrome patients carried missense mutations within the NR2B ion channel-forming re-entrant loop (p.Asn615Ile, p.Val618Gly). Subsequent screening of 47 patients with unexplained infantile spasms did not reveal additional de novo mutations, but detected a carrier of a novel inherited *GRIN2B* splice site variant in close proximity (c.2011-5_2011-4delTC). Mutations p.Asn615Ile and p.Val618Gly cause a significantly reduced Mg^2+^ block and higher Ca^2+^ permeability, leading to a dramatically increased Ca^2+^ influx, whereas p.Arg540His caused less severe disturbance of channel function, corresponding to the milder patient phenotype.

**Interpretation:**

We identified *GRIN2B* gain-of-function mutations as a cause of West syndrome with severe developmental delay as well as of ID with childhood onset focal epilepsy. Severely disturbed channel function corresponded to severe clinical phenotypes, underlining the important role of facilitated NMDA receptor signaling in epileptogenesis.

Epileptic encephalopathies (EEs) constitute a group of disorders in which the epileptic activity itself is considered to contribute to severe cognitive impairment or decline above and beyond what might be expected from the underlying pathology alone.^1^ West syndrome belongs to this heterogeneous group of disorders presenting with distinctive clinical and electrophysiological features usually manifesting between 3 and 12 months as clusters of infantile spasms (IS) and a characteristic electroencephalogram (EEG) pattern called hypsarrhythmia.^2^ The etiology of West syndrome is very diverse, and a substantial subgroup is considered to have a genetic origin. West syndrome has been associated with mutations in *ARX*, *CDKL5*, *STXBP1*, and *ST3GAL3* as well as various copy number variations (CNVs).^3^–^7^ However, in many cases the genetic defect remains unresolved.

Mutations in *GRIN2A* and *GRIN2B* encoding the alpha and beta-2 subunits (NR2A and NR2B) of the glutamate-activated N-methyl-D-aspartate (NMDA) receptor are associated with several neurodevelopmental disorders. Mutations in *GRIN2A* have recently been detected in idiopathic focal epilepsy with rolandic spikes and related epileptic encephalopathies, that is, in Landau–Kleffner syndrome, epilepsy with continuous spike-and-waves during slow sleep syndrome, and nonsyndromic epilepsy associated with intellectual disability (ID).^8^–^11^ By contrast, *GRIN2B* has not been described as an epilepsy gene to date but has repeatedly been considered as a putative candidate gene for seizures,^8^,^12^ and mutations were detected in patients with ID, autism spectrum disorders (ASD), and schizophrenia.^8^,^13^–^18^

## Materials and Methods

We used a targeted massive parallel resequencing approach to diagnostically screen 357 individuals (Cohort A) with a broad range of epilepsy phenotypes. The panel contained 50 known genes comprising EE genes, plus genes for severe ID not associated with seizures, but nevertheless suspected to be involved in epileptogenesis (eg, voltage-sensitive and ligand-gated ion-channel genes). Analysis was performed as described previously.^19^ Ninety-one of the 357 individuals were diagnosed with EE. Detailed clinical information necessary for a more specific epilepsy syndrome classification was not available for many of these 91 patients. After detecting de novo mutations in *GRIN2B* within Cohort A, we subsequently screened 47 patients with unexplained IS (Cohort B) by conventional methods. Three patients were diagnosed with Ohtahara syndrome and 38 with West syndrome, and 6 patients had a nonsyndromic early onset EE with IS during the course of the disease.

### Sequence Analysis

We performed direct Sanger sequencing to detect point mutations/small indels as well as multiplex amplicon quantification (MAQ) to detect CNVs in DNA extracted from peripheral blood. All 13 exons and intron–exon boundaries of *GRIN2B* were analyzed by bidirectional sequencing with the BigDye Terminator v3.1 Cycle Sequencing kit on an ABI3730 DNA Analyzer (Applied Biosystems, Foster City, CA; primers available upon request).

Additionally, the genomic region containing *GRIN2B* was screened for CNVs by use of an in-house–developed technique for MAQ (http://www.multiplicom.com/multiplex-amplicon-quantification-maq) in Cohort B. This assay comprises a multiplex polymerase chain reaction (PCR) amplification of fluorescently labeled target and reference amplicons, followed by fragment analysis on the ABI3730 DNA Analyzer.^20^ The comparison of normalized peak areas between the test individual and the average of 5 control individuals results in the target amplicon doses indicating the copy number of the target amplicon (using the in-house–developed Multiplex Amplicon Quantification Software; http://www.multiplicom.com/maq-s). The multiplex PCR reaction consists of 10 test amplicons located in the genomic region of *GRIN2B* and 6 reference amplicons randomly located on different chromosomes (primer mix is available upon request).

### In Silico Prediction

Pathogenic implications of identified coding variants were assessed by different in silico analysis programs (PolyPhen2, http://genetics.bwh.harvard.edu/pph2/ and MutationTaster, http://www.mutationtaster.org; Table 1). For intronic single nucleotide polymorphisms, splice site analysis was performed using HSF2.4 (http://umd.be/HSF/).

**Table 1:** Mutations Detected in *GRIN2B*

Patient	Phenotype	Mutation	Prediction (MutationTaster/Polyphen-2)	Origin	Domain	Functional Effect
1	West syndrome	c.1853T>G, p.Val618Gly	Disease causing/damaging	De novo	Channel pore	Gain of function
2	West syndrome	c.1844A>T, p.Asn615Ile	Disease causing/damaging	De novo	Channel pore	Gain of function
3	Focal epilepsy & ID	c.1619G>A, p.Arg540His	Disease causing/damaging	De novo	Glutamate-binding domain	Gain of function (mild)
4	West syndrome	c.2011-5_2011-4delTC	Not applicable	Paternal	Not applicable	Potential splice defect

ID = intellectual disability.

The mutation of the ligand-binding domain in *GRIN2B* encoding human NR2B was analyzed using the x-ray crystal structure^21^ of rat NR2A (Protein Data Bank # 2A5S). Rat NR2A and human NR2B share 82% sequence identity and 88% sequence similarity in their glutamate-binding regions, making the rat NR2A structure a useful template for the analysis of mutations in *GRIN2B*. The interactive visualization program University of California, San Francisco (UCSF) Chimera^22^ was used for structural analysis. The “swapaa” command was used to substitute amino acids, selecting the side chain from the Dunbrack backbone-dependent rotamer library based on lowest number of clashes, highest number of hydrogen bonds, and highest probability.

Molecular modeling of the transmembrane domains of NR1/NR2B receptors was based on the crystal structure of GluR2 (Brookhaven Protein Data Bank entry 3KG2) using Modeller 9v6 (UCSF Sali Lab) and lsqman 9.7.9 (Uppsala Software Factory, Uppsala, Sweden) as described.^21^ Models were subjected to short-term molecular dynamics simulations using the Charmm27 force field, which is implemented in the Tinker 4.2 molecular modeling software (http://dasher.wustl.edu/tinker/). Figures were made using PyMOL 1.2 (http://www.pymol.org).

### Functional Investigations

For *Xenopus laevis* oocyte experiments, NR1-1a and NR2B constructs and capped cRNAs were generated as described previously.^8^ Mutations were introduced into these constructs using the QuikChange site-directed mutagenesis kit (Stratagene, Agilent Technologies, Santa Clara, CA) and confirmed by Sanger DNA sequencing. Individual stage V to VI oocytes were obtained from anaesthetized frogs and isolated by collagenase treatment. Ten nanograms of total NR1/NR2B cRNA were injected into oocytes. Following injection, oocytes were kept at 17°C in ND96 solution (96mM NaCl, 2mM KCl, 1.8mM CaCl_2_, 1mM MgCl_2_, 5mM HEPES, pH 7.4). Glutamate and glycine dose–response curves of wild-type NR1/NR2B and mutant NMDA receptors were analyzed by 2-electrode voltage-clamp recording as described.^23^ Concentration–response curves and current traces shown in the figures were drawn using KaleidaGraph (Synergy Software, Reading, PA). To monitor the voltage dependence of NR1-NR2B receptor combinations, whole-cell current-voltage relationships of saturating glutamate- and glycine-induced currents were recorded in 20mV intervals ranging from −90mV to +30mV and normalized to the current value obtained at +30mV above the respective reversal potential as described previously.^24^ The relative divalent to monovalent permeability was calculated by the Goldman–Hodgkin–Katz constant field voltage equation assuming no anion permeability. The internal concentrations of Na^+^ and K^+^ used in the calculations were 20mM and 150mM, respectively.^24^ Permeability ratios were calculated for each oocyte and then averaged. Mg^2+^ inhibition (1mM) was evaluated in the presence of 1.8mM Ca^2+^ at a holding potential of −70mV upon application (5 seconds) of saturating glycine (10μM) and glutamate (100μM) concentrations.

### Statistical Analyses

Values given represent means ± standard error of the mean. Statistical significance was determined at the *p* < 0.05, *p* < 0.01, and *p* < 0.001 levels using Student 2-tailed, unpaired *t* test.

## Results

### Sequence Analysis and Functional Investigations

**Figure 1:**
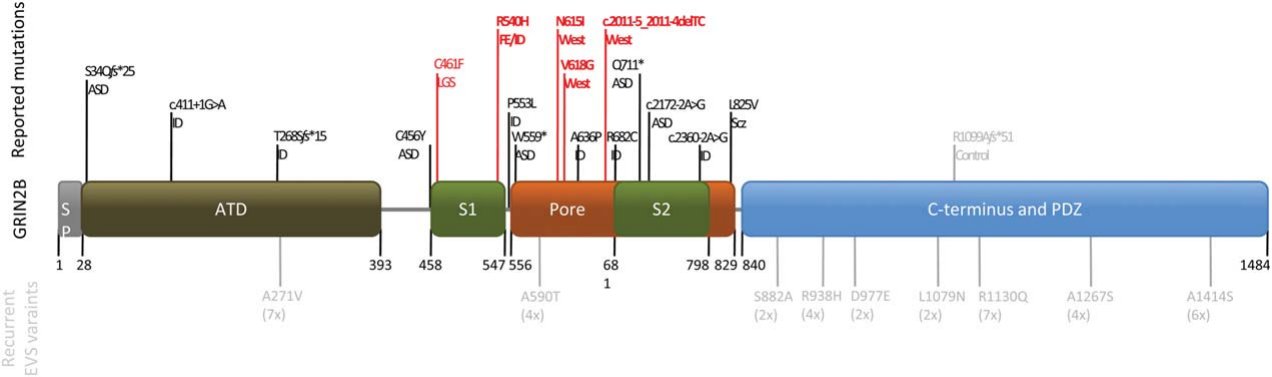
Location of *GRIN2B* mutations in a schematic illustration of the conserved domains of the NR2B subunit (SP = signal peptide; ATD = amino-terminal domain, involved in receptor assembly; S1 and S2 form the ligand-binding domain; Pore = re-entrant pore-forming and transmembrane spanning domains; PDZ = PDZ domain binding motif). All reported de novo mutations and their according phenotypes (ASD = autism spectrum disorders; FE = focal epilepsy; ID = intellectual disability; LGS = Lennox–Gastaut syndrome; Scz = schizophrenia) are listed in the top row. Mutations causing phenotypes without seizures are labeled in black, mutations in epilepsy patients are in red. So far, no pathogenic variants have been observed in the C-terminal region of NR2B. Mutations causing West syndrome cluster within re-entrant pore-forming domain, whereas the mutation causing ID and focal epilepsy was observed in the glutamate-binding domain S1, similar to a recently described LGS case. Nonsynonymous variants that are believed not to be associated with abnormal phenotypes (gray) and are reported more than once (in brackets) in the Exome Variant Server (EVS) are listed in the bottom line.

Among the 357 patients of Cohort A, we identified 2 individuals with West syndrome (Patients 1 and 2) carrying novel heterozygous de novo mutations in *GRIN2B* (2 of 91 EE cases, 2.2%) affecting key amino acids (p.Val618Gly and p.Asn615Ile) within the NR2B ion channel-forming re-entrant loop, as well as a patient with ID and childhood onset focal epilepsy (Patient 3) carrying a novel heterozygous de novo mutation (p.Arg540His) within the NR2B glutamate-binding domain (see Table, Fig 1).

**Figure 2:**
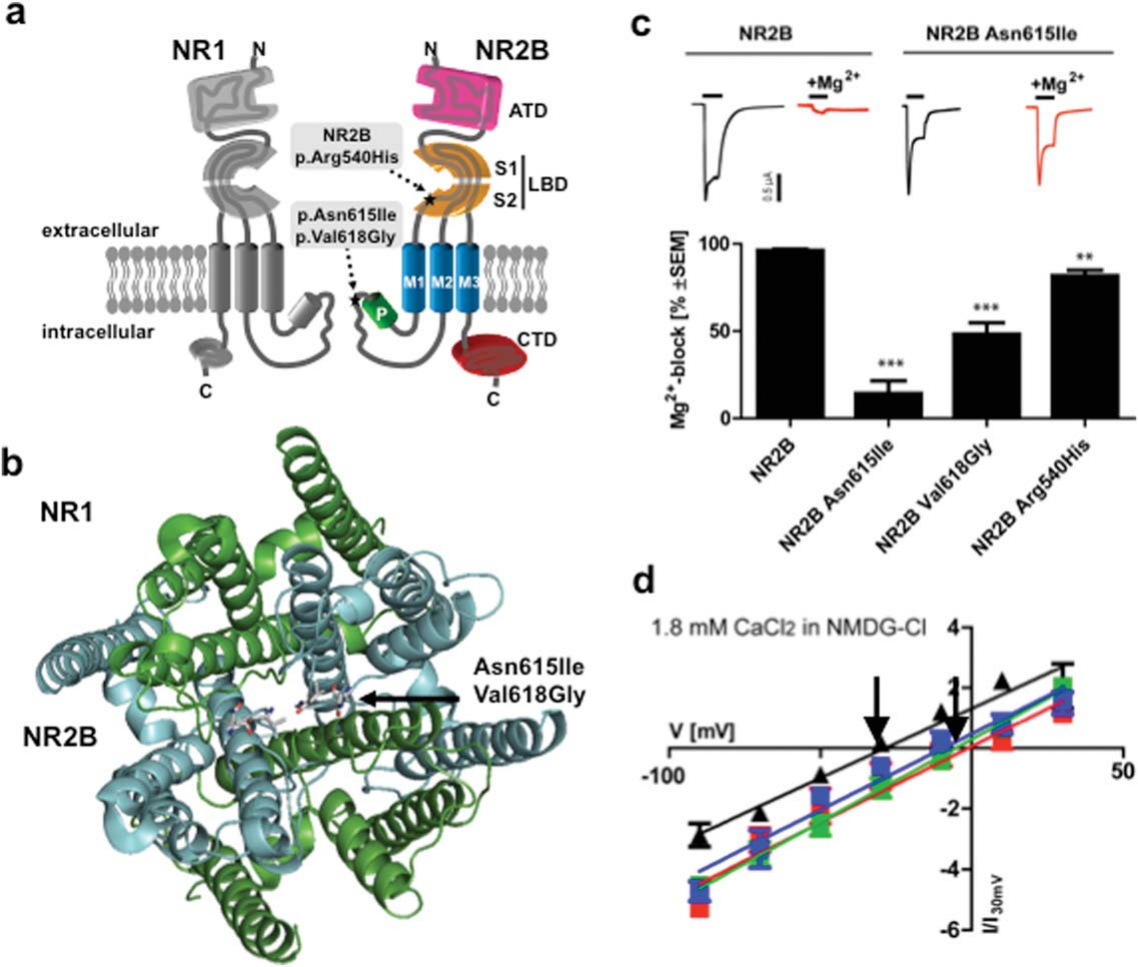
Structural and functional consequences of missense mutations in *GRIN2B*. (**A**) Topology model of an NR1 and an NR2B subunit. Positions of the alterations p.Arg540His, p.Asn615Ile and p.Val618Gly are indicated by asterisks in the NR2 subunit consisting of an amino-terminal domain (ATD), the ligand-binding domain (LBD) including the S1 and S2 peptide segments, 3 transmembrane segments (M1, M2, and M3), a re-entrant pore loop (P), and an intracellular carboxy-terminal domain (CTD). Residue Arg540 lies within the glutamate-binding domain, and Asn615 and Val618 in the ion channel pore. N = NH_2_-terminus; C = COOH-terminus. (**B**) Model of the transmembrane arrangement of the N-methyl-D-aspartate (NMDA) receptor composed of NR1 (green) and NR2B (cyan) subunits (top view). The arrow highlights the side chains of p.Asn615Ile and p.Val618Gly in the pore-forming region. (**C**) Gradual loss of Mg^2+^ inhibition of NR1-NR2B wild-type and NR1-NR2B mutant receptor currents at −70mV. Respective sample traces of NR1-NR2B and NR1-NR2B^Asn615Ile^ are shown above with inhibition of receptor currents by 1mM Mg^2+^ of NR1-NR2B (96 ± 0.9%, n=4) and mutant NR1-NR2B^Asn615Ile^ (14 ± 7.2%, *p* < 0.0001, n = 3), NR1-NR2B^Val618Gly^ (48 ± 6.5%, *p* = 0.0003, n = 3), and NR1-NR2B^Arg540His^ (81 ± 3.2%, *p* = 0.005, n = 5) receptors. (**D**) Effect on Ca^2+^ permeability of NR1-NR2B wild-type and NR1-NR2B mutant receptor currents. Current–voltage relationships of NR1-NR2B receptors in the absence of Mg^2+^ in Na^+^-free extracellular solution reveal significant differences in the reversal potential (indicated by *arrows*) of NR1-NR2B (−31 ± 1.7mV, n = 4, *black triangles*) and mutant NR1-NR2B^Asn615Ile^ (−1.0 ± 6.8mV,*p* = 0.004, n = 3, *red squares*), NR1-NR2B^Val618Gly^ (−5.4 ± 3.7mV, *p* < 0.001, n = 3, *green squares*), and NR1-NR2B^Arg540His^ (−9.4 ± 6.5mV, *p* = 0.013, n = 3, *blue squares*) receptor currents. (NMDG-Cl, N-methyl-D-glucamine chloride) Calculation of the relative divalent to monovalent cation permeability *P*Ca/*P*Na by the Goldman–Hodgkin–Katz voltage equation revealed a >3-fold increase in Ca^2+^ permeability of the mutant NMDA receptors (*P*Ca/*P*Na for NR1-NR2B = 0.86; NR1-NR2B^Asn615Ile^ = 5.22; NR2B^Val618Gly^ = 3.12; and NR2B^Arg540His^ = 3.23).

**Figure 3:**
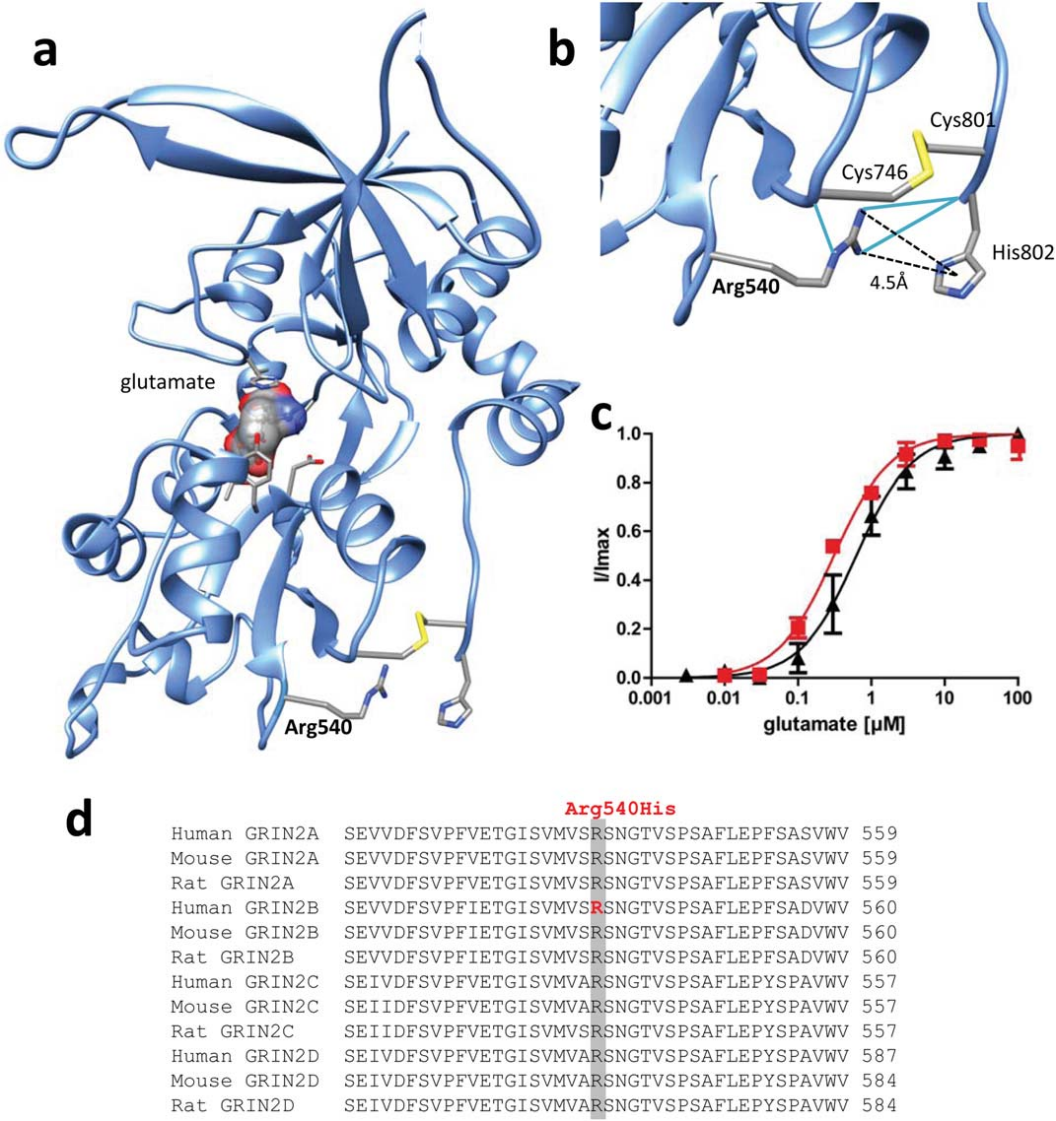
Structural and functional analyses of the glutamate binding-domain mutation Arg540His. (**A**) Residue 540 is predicted to be located within the glutamate-binding S1 domain, and is significant in the stabilization of the tertiary structure of the glutamate-binding domain. (**B**) Substitution p.Arg540His is likely to abolish hydrogen bonding *(blue lines)* with the backbone of Cys746 and His802 and a cation–pi interaction with His802, possibly leading to a relaxed fold in this region. (**C**) Pharmacological characterization of the apparent agonist affinities of wild-type NR1-NR2B *(black triangles)* and mutant NR1-NR2B^Arg540His^ *(red squares)* N-methyl-D-aspartate receptors measured after heterologous expression in *Xenopus laevis* oocytes by 2-electrode voltage-clamping revealed that similar glutamate concentrations were required to elicit half-maximal responses (EC_50_ values = 0.72 ± 0.22μM and 0.31 ± 0.02μM, respectively, *p* = 0.14, n = 3). (D) Arg540 is a highly conserved residue within NR2A–D subunits.

Mutation p.Asn615Ile (Patient 2) affects 1 of 2 paired asparagines (Asn615, Asn616 in NR2B) in the re-entrant pore-forming loop implicated in Mg^2+^ block, and p.Val618Gly (Patient 1) is in close vicinity. Expression of NR1/NR2B^Asn615Ile^ and NR1/NR2B^Val618Gly^ heteromers revealed a significant loss of ion-channel block by extracellular Mg^2+^ and a dramatically increased Ca^2+^ permeability (Fig 2), consistent with a gain of function and consequent hyperexcitability. By contrast, p.Arg540His (Patient 3) affects a highly conserved residue located in the extracellular glutamate-binding region. Mutation of p.Arg540His is predicted to abolish the hydrogen bonding with the backbone of Cys746 and His802, and a cation–pi interaction with His802, possibly leading to a relaxed fold in this region (Fig 3). Curiously, rather than affecting glutamate binding, p.Arg540His also resulted in a decrease of Mg^2+^ block and increase of Ca^2+^ permeability, implying an allosteric effect for this mutation. However, the functional impacts were less severe, in line with the milder phenotype of the patient (see Fig 2).

Among the 47 patients of Cohort B, we detected 1 additional West syndrome patient (Patient 4) with a novel paternally inherited heterozygous splice-site variant deleting 2 base pairs of the splice acceptor site of exon 10, which encodes parts of the ion-channel pore domain of *GRIN2B*. In silico prediction suggests a consecutive alternative splice acceptor site 6 base pairs downstream of the mutated splice site that is only marginally weaker compared to the wild-type splice acceptor site. Use of this alternative splice acceptor site is predicted to result in an in-frame deletion of amino acids Phe671 and Gln672 (p.Phe671_Gln672del) of the *GRIN2B* pore complex, which would be in line with the location of the mutations of the 2 West syndrome patients described above. However, the latter putative splice variation was inherited from the patient’s healthy father, and no fresh sample or tissue was available to functionally confirm the potentially aberrant splicing. Screening for copy number variations by MAQ did not reveal additional deletions or duplications affecting *GRIN2B* in Cohort B.

### Patient Phenotypes

Patient 1 was a male born at term after an uneventful pregnancy. Myoclonic jerks and infantile spasms occasionally occurring in clusters at the age of 4 months led to the diagnosis of West syndrome. At 6 months old, he held no eye contact and presented with episodic hyperextension of axial muscles. The result of first EEG was not available for review. EEG at age 8 months showed multifocal bursts of irregular spike waves as well as rhythmic bilateral generalized spike waves with a frequency of 4 to 5 per second reminiscent of modified hypsarrhythmia. During sleep, there was rare irregular high-amplitude epileptiform activity with a hypsarrhythmialike pattern. Treatment with vigabatrin and pyridoxine led to a slight clinical improvement. Replacement of vigabatrin by levetiracetam improved neither seizures (predominantly of myoclonic type) nor EEG pattern, whereas implementation of valproate finally led to a significant reduction of seizure frequency. At last follow-up at age of 2 years and 1 month, the boy’s length was at P90, weight at P25, and head circumference at P50. He had severe axial hypotonia with episodic hyperextension and could not sit independently. He could hold eye contact only briefly before drifting away. At this time, the boy presented additionally with dystoniclike movements of his fingers and still had no verbal communication.

Patient 2 was a female born at term after an uneventful pregnancy. At the age of 7 weeks, she presented with infantile spasms. She held no eye contact and showed muscular hypotonia. She had episodic hyperextension of axial muscles. These episodes were not recorded on video EEG, and an epileptic nature could not be excluded. Brain magnetic resonance imaging (MRI), magnetic resonance spectroscopy, cerebrospinal investigation, and metabolic workup were normal. EEG showed typical hypsarrhythmia. Treatment with vigabatrin, sulthiame, and topiramate failed to decrease seizures, whereas implementation of steroid pulse therapy led to an improvement. At last follow-up at age 5 years and 3 months, the girl is not able to sit independently. She presented with autisticlike behavior and no verbal expression, severe feeding difficulties, and mild microcephaly. The EEG showed increased theta activity without epileptic discharges. The girl still exhibits series of epileptic spasms as well as occasional generalized tonic–clonic seizures.

Patient 3 was a female born at 41 weeks after an uneventful pregnancy and conception by in vitro fertilization. Early development was delayed, as she sat at 11 months, walked at 19 months, and used her first words at 18 months. At the age of 3 years, she was diagnosed with global developmental delay. There was no evidence for stagnation or even regression of development. Since the age of 9 years and 9 months she had focal dyscognitive seizures with postictal paresis of the right arm as well as occasional bilateral convulsive seizures and status epilepticus. Postictal EEG showed slowing over the left frontoparietal region, and MRI of the brain showed postictal diffusion restriction in the same region that resolved over time. Lumbar puncture and metabolic screening were normal. At the last follow-up at the age of 10 years and 6 months she had a mild intellectual disability and occasional seizures with postictal paresis.

Patient 4 was a male born at term after an uneventful pregnancy. At the age of 2 months epileptic spasms occurred, which soon evolved into asymmetric tonic seizures. EEG at the age of 7 months showed hypsarrhythmia, and multifocal epileptic activity was seen at the age of 14 months. MRI of the brain, metabolic workup, and lumbar puncture were all normal. At last follow-up at the age of 4 years, he still had daily therapy-resistant tonic seizures and focal motor seizures. He had severe intellectual disability with hypotonic muscle tone and was unable to talk or walk.

## Discussion

Our investigations revealed de novo *GRIN2B* missense mutations in 2 of 91 patients with unexplained EE (2.2%) and in 1 patient with ID and childhood onset focal epilepsy. We also detected 1 novel inherited putative splice variant in 1 of 47 patients with IS. The observation of a possibly reduced penetrance of an inherited splice variant in Patient 4 is in agreement with previous observations^9^ in families carrying mutations and putative splice variants in *GRIN2A*. However, much larger data sets are needed to prove a role of inherited *GRIN2B* variants as risk factors for these disorders.

Interestingly, the very recent study of the Epi4K consortium revealed 1 additional case of a de novo mutation in *GRIN2B* in 1 of 115 individuals with epileptic encephalopathy of Lennox–Gastaut type (see Fig 1).^12^ The patient shows parallels to Patient 3 of our present study. Both mutations p.Arg540His (Patient 3) and p.Cys461Phe (Epi4K patient) are located in the extracellular glutamate-binding region, and both patients presented with primary developmental delay and childhood onset epilepsy.

NMDA receptors are tetrameric ligand-gated ion channels permeable to Na^+^, K^+^, and Ca^2+^, composed of 2 glycine-binding NR1 subunits and 2 glutamate-binding NR2 subunits (NR2A, NR2B, NR2C, NR2D).^25^,^26^ Subunit composition of NMDA receptors is spatially and temporally regulated with a switch from predominant NR2B expression in early development to more prominent synaptically localized NR2A expression at later stages,^27^,^28^ which might explain the tendency toward earlier onset epilepsy phenotypes in *GRIN2B* versus *GRIN2A* mutation carriers.

NMDA receptor subunits are organized into multiple structural domains (see Fig 1) including a signal peptide, an amino-terminal domain involved in receptor assembly, S1 and S2 segments that form the ligand binding domain, 3 membrane-spanning domains M1–M3, and a re-entrant pore-forming loop. Lastly, a large intracellular C-terminus and PDZ domain-binding motif mediate interactions with intracellular proteins such as PSD95. In the Exome Variant Server (EVS; National Heart, Lung, and Blood Institute Exome Sequencing Project, http://evs.gs.washington.edu/EVS/), 38 missense and no putative essential splice variants are listed in *GRIN2B* in 6503 healthy controls. The described variants are unevenly distributed, with the vast majority (27 of 38) of variants being positioned within the NR2B C-terminus. Only 9 of 38 variants were detected repeatedly, and again 7 of these are within the C-terminus. Curiously, the only described de novo aberration within the NR2B C-terminus was detected in an apparently healthy control subject,^29^ whereas all known pathogenic *GRIN2B* mutations causing neurodevelopmental disorders are found within the N-terminal region, ligand-binding S1 and S2 segments, and the re-entrant pore-forming loop (see Fig 1).^8^,^13^–^18^ This suggests that pathogenic mutations affecting key functional motifs have a greater impact on protein function and can negatively influence neurodevelopment and brain excitability. This is also reflected by the finding that variants outside the C-terminus of *GRIN2B* occurred significantly more frequently in alleles of EE individuals of Cohorts A and B compared to the EVS controls (*p* = 0.0027, Fisher exact test).

Interestingly, 2 of the West syndrome patients presented gain-of-function mutations (Patient 1, p.Val618Gly and Patient 2, p.Asn615Ile) in the re-entrant pore-forming loop. By contrast, Patient 3 with ID, childhood onset focal epilepsy, and less severe developmental delay carried a milder gain-of-function mutation (p.Arg540His), positioned in the extracellular glutamate-binding S1 domain. Additionally, in support of the hypothesis that epilepsy is caused by gain-of-function mutations in *GRIN2B*, truncating and thus predicted loss-of-function mutations have only been described in patients with ID and/or ASD so far.^18^ These data suggest a distinct genotype–phenotype correlation, although more mutations and functional studies are needed to verify this assumption.

In summary, we postulate that genetic alterations in *GRIN2B* are responsible for ∼2% (2 of 91) of EE cases, preferentially causing IS and West syndrome. This further strengthens the concept that West syndrome comprises a heterogeneous group of several disease entities, causing increased brain excitability with a similar and age-related EEG and seizure pattern. Additionally, our findings reveal further evidence of the contribution of altered NMDA receptor signaling to epileptogenesis and establish *GRIN2B* as another key player in epileptic encephalopathies. In the patients described above, the severity of phenotypes corresponds to the electrophysiological severity of gain of ion channel function. This is of particular interest as existing NMDA receptor blockers such as memantine represent promising drugs to selectively restore the altered NMDA receptor function in patients with gain-of-function mutations in NR2 subunits, putting NMDA receptors more in focus in the search for new targets in epilepsy treatment.^30^
